# An Artificial Intelligence-Assisted Smartphone Application for Improving Dietary Quality Among Frail Older Adults: A Quasi-Experimental Study

**DOI:** 10.3390/geriatrics10060160

**Published:** 2025-12-04

**Authors:** Kayo Kurotani, Hikaru Tanabe, Keiji Yanai, Kazunori Sakamoto, Kazunori Ohkawara

**Affiliations:** 1 Graduate School of Life Sciences, Showa Women’s University, Tokyo 154-8533, Japan; 2 Graduate School of Informatics and Engineering, The University of Electro-Communications, Tokyo 182-8585, Japan; 3 Faculty of Information and Management, Tokyo Online University, Tokyo 160-0023, Japan

**Keywords:** AI, app, ICT, intervention, social connection, diet diversity, loneliness, frail, older adults

## Abstract

**Background/Objectives**: Although information and communication technology (ICT) offers opportunities to address challenges, evidence among frail populations is limited. We aimed to evaluate the effectiveness and feasibility of an ICT-based intervention incorporating an artificial intelligence (AI)-assisted smartphone dietary application and group communication tools to improve dietary quality and social connection among community-dwelling older adults with frailty. **Methods**: A non-randomized, quasi-experimental study was conducted among 29 older adults (≥65 years) in Tokyo, Japan. Participants were assigned to the intervention (*n* = 11) or control (*n* = 18) group. The 3-month intervention included weekly photo uploads of meals via an AI-based dietary application providing automated image analysis and personalized feedback, supervised by registered dietitians, along with peer communication through a group chat. The primary outcome was dietary quality. The secondary outcomes included body weight, body mass index (BMI), skin carotenoid score, and loneliness. **Results**: The adjusted Japanese Food Guide Spinning Top Score at 3-month follow-up was 49.0 (standard error [SE] = 2.6) and 39.5 (SE = 2.0) in the intervention and control groups, respectively. The adjusted mean difference between groups was +9.5 (95% confidence interval: 2.3 to 16.7, *p* = 0.01). After using analysis of covariance for adjusting for respective baseline values, age, education status, and antihypertension drug use, no statistically significant between-group differences were observed at 3-month follow-up for any secondary outcomes. **Conclusions**: AI-based dietary intervention and peer communication effectively improved dietary quality among older adults, highlighting the potential of such an intervention to promote healthier eating habits in this population.

## 1. Introduction

Frailty is a multidimensional geriatric syndrome associated with disability, hospitalization, and mortality. Among its modifiable determinants, dietary quality plays a central role [1]. Dietary variety is an important determinant of overall food intake [2]. Higher dietary variety scores (DVSs) are associated with lower risks of malnutrition, functional decline, and mortality among older adults [3,4,5]. The DVS is a food-based composite index calculated from the consumption frequencies of 10 food groups, meat, fish/shellfish, eggs, milk, soybean products, green/yellow vegetables, potatoes, fruits, seaweed, and fats/oils, which together constitute a substantial proportion of typical Japanese main and side dishes [3]. A cluster-randomized controlled trial encouraging higher dietary variety improved dietary behaviors and promoted physical activity among community-dwelling older adults in Japan [6]. Although these associations are well documented, strategies to effectively enhance dietary variety among older adults with frailty remain unclear.

Recently, the World Health Organization Commission on Social Connection has published a report emphasizing that social connection is essential for the health, strength, and resilience of individuals and societies [7]. Policy-level attention to loneliness has also increased, with the United Kingdom appointing a Minister for Loneliness in 2018 and Japan following in 2021 [8]. Social isolation and loneliness are major risk factors for mortality, with effect sizes comparable to those of smoking or obesity [9]. A systematic review and meta-analysis published in 2022 reported significant cross-sectional and longitudinal associations between loneliness and frailty in community-dwelling older adults [10]. In Japan, recent longitudinal evidence indicates that older adults who are socially isolated or lonely have a markedly increased risk of developing frailty compared with socially connected individuals [11]. Beyond individual dietary habits, accumulating evidence highlights a critical interrelationship between dietary quality and social connectedness, particularly in older adults [12]. Poor nutritional status, characterized by inadequate intake of several nutrients, can negatively affect mental health [13]. Conversely, social isolation has been associated with malnutrition in older urban adults in Japan [14]. Therefore, interventions that address both nutritional intake and opportunities for social engagement may hold promise in leveraging these shared mechanisms, such as reduced appetite, decreased motivation for cooking, and difficulties in grocery shopping, which are often exacerbated by both poor nutrition and social isolation, to achieve more holistic health improvements. Despite these established associations, effective interventions targeting socially vulnerable frail adults remain scarce.

Recent advances in information and communication technology (ICT) have provided new opportunities to address poor dietary quality and social disconnection. A recent meta-analysis reported that smartphone applications could improve healthy eating behaviors, although the included studies did not involve older adults [15]. According to the Cabinet Office (2023) Public Opinion Survey on the Use of Information and Communication Devices, almost all adults aged <60 years reported using smartphones or tablets, whereas the proportion declined to 84.2% among those in their 60s and to only 48.4% among those aged ≥70 years [16]. Thus, older adults experience a digital divide, and the effectiveness of smartphone applications, devices that are familiar and widely used among younger populations, remains insufficiently understood in older populations. While various digital tools can help with dietary assessment and behavior change, artificial intelligence (AI) offers particular advantages for interventions targeting older adults. Unlike labor-intensive manual logging or simpler digital methods, AI-driven image analysis provides objective, real-time dietary feedback with minimal user burden [17,18,19]. For older populations, this is valuable as it simplifies the assessment process, provides personalized, actionable insights, and can enhance engagement without requiring extensive digital literacy. This distinctive efficiency and personalization offer significant advantages over conventional digital tools, potentially leading to improved adherence and sustained behavioral change. Although one review reported that AI functionalities in nutrition applications remain relatively rare and that only a small number of studies specifically targeted older adults [20], another highlighted the growing number of AI-based dietary applications worldwide, with their validity and limitations increasingly being examined [21]. Several applications have also been tested as intervention tools and have shown potential for improving dietary behaviors in various populations [22]. Despite these developments, there remains a notable paucity of studies focusing on comprehensive AI-driven dietary interventions, particularly those integrating expert oversight and social support for improving dietary quality in older adults. Such a comprehensive strategy targeting community-dwelling frail older adults requires further investigation to explore its feasibility and preliminary effectiveness. Nevertheless, few studies have specifically targeted older adults [23], and to the best of our knowledge, none have focused on older adults with frailty.

Thus, this study aimed to evaluate the effectiveness of an AI-powered smartphone application intervention, focusing on dietary quality as the primary outcome and social connection as the secondary outcome among community-dwelling older adults with frailty.

## 2. Materials and Methods

### 2.1. Study Design and Participants

This quasi-experimental intervention study was conducted among community-dwelling older adults aged ≥65 years residing in the municipality of Tokyo, Japan. The exclusion criteria were as follows: (1) currently under medical or dietary treatment with physician or dietitian guidance and (2) unable to perform basic smartphone operations independently (e.g., entering text, taking photographs). Thirty individuals consented to participate, one of whom withdrew before the intervention, leaving 29 participants for the analysis. The participants were assigned to either the intervention (*n* = 11) or control (*n* = 18) groups. Staff at the community space where older adults gathered were asked to recruit participants assign them accordingly. Individuals with higher smartphone proficiency were allocated to the intervention group. In addition, participants who were unable to attend the baseline assessment scheduled for the intervention group were subsequently assigned to the control group. Baseline assessments were conducted in December 2024, and follow-up surveys were conducted in March 2025. Frailty status was assessed using the Kihon Checklist (KCL), with a score of ≥8 defined as frailty [24,25]. In addition, the participants completed a brief self-administered questionnaire on lifestyle and health status and a dietary survey using the Brief-type Self-administered Diet History Questionnaire (BDHQ). At baseline, all participants met the frailty criteria. All 29 participants completed both the baseline and follow-up assessments.

This study was approved by the Ethics Committee of Showa Women’s University (approval no. 24-14). The current study was registered in the University Hospital Medical Information Network Clinical Trials Registry (UMIN-CTR; UMIN000056396; registration date: 8 December 2024) as a registered trial.

### 2.2. Intervention

Participants assigned to the intervention group were further divided into small subgroups of 4–7 members. The program began with an in-person session, which included the following: (1) education on the DVS based on 10 food groups, encouraging participants to aim for ≥7 groups daily; (2) demonstration of a balanced meal using a standardized “Smart Meal” [26] boxed lunch; (3) training on the use of the newly developed *Shokuji Kōkan Nikki* (SKN) (Dietary Exchange Diary) smartphone application; (4) practice in taking food photos using the Smart Meal lunch.

Over the following 3 months, the participants used the SKN application to upload photos of all meals on one designated day per week. Team members could view each other’s photos and post reactions and comments, thereby facilitating peer interactions. The application incorporated an AI (ChatGPT-based) image analysis module that estimated from photos the 10 DVS groups contained in the meal, and assessed and created feedback on the strengths and areas for improvement per breakfast, lunch, and dinner. It also comprehensively estimated the DVSs consumed across all daily meals to generate day by day evaluations and advice.

All AI-generated evaluations and advices were confirmed by a registered dietitian; any errors were corrected, and if no corrections were needed, the feedback was directly provided to the participants. Furthermore, the dietitian integrated individual results within each group to calculate the average DVS consumed per day by group members and created group-specific advice. Notably, the dietitian had minimal direct communication with the participants, with peer-to-peer interactions among participants forming the primary mode of communication.

Findings from our pilot validation study indicated that AI recognition accuracy was the highest for photos taken from above and improved when ingredient details were provided. Therefore, the participants were instructed to photograph meals from above whenever possible and provide supplementary descriptions of dish ingredients using the application’s input function. These instructions were incorporated into the user manual.

In addition, small groups were provided with Smart Meals only during the initial in-person session to support practical dietary learning. Furthermore, a group chat was separately established from the application to allow informal communication, in which the participants exchanged information about local food availability and asked questions.

### 2.3. Validity of Artificial Intelligence (AI) Image Analysis

The AI-based image analysis system (GPT-4o, OpenAI) classified foods into the 10 groups of the DVS. Its validity was preliminarily tested in this pilot study, in which the AI-based classification of 65 dishes (260 images across four angles) was compared with reference values. These 65 dishes were carefully selected as culturally typical mixed meals commonly consumed by Japanese people. The reference values were established by registered dietitians who strictly adhered to recipes during the preparation of the photographed dishes. This method ensured that the nutritional values derived from the recipes were highly accurate and served as a robust test of the AI’s performance. The overall agreement rate was 83%, with the highest accuracy observed for milk/dairy products and the lowest for fats/oils. The accuracy tended to be higher for images taken from above, whereas mixed dishes, such as stews or fried rice, were more challenging. These results from this preliminary assessment indicate the acceptable validity of the system, and registered dietitians supervised and corrected all feedback to ensure accuracy. Based on these findings, the participants were instructed to take photos whenever possible and provide supplementary descriptions of dish ingredients using the application’s input function. These instructions were included in the user manual to enhance the accuracy of AI-based classification.

### 2.4. Control Group

The control group did not receive any intervention during the study period. Gift cards were provided as incentives to promote retention and follow-up participation.

### 2.5. Outcome Measures

The primary outcome was dietary balance, assessed using the Japanese Food Guide Spinning Top Score, which quantifies adherence to the Japanese Food Guide Spinning Top. The score ranges from 0 to 70, with higher scores indicating greater adherence to the recommended dietary balance [27]. The score is calculated based on the number of servings of grain dishes, vegetable dishes, fish and meat, milk and dairy products, and fruits and the total energy intake and energy from confectioneries, alcoholic beverages, and sugar-sweetened beverages.

The secondary outcomes included loneliness, body weight, body mass index (BMI), and skin carotenoid score. Loneliness was measured using the three-item UCLA Loneliness Scale, a validated short form consisting of three questions (e.g., “How often do you feel left out?”). There are four choices per item: (1) never, (2) rarely, (3) sometimes, and (4) always. Higher scores indicate a higher level of loneliness [28]. The Japanese version has been validated among older adults [29,30]. Body weight and height were measured following standard protocols (barefoot and standing upright) using a calibrated wall-mounted stadiometer and scale, with precision to 0.1 cm and 0.1 kg, respectively. Because all measurements were conducted in winter with the participants wearing typical indoor clothing (e.g., sweaters, thick pants), clothing weight corrections were applied. These specific correction values, −0.8 kg for women and −1.2 kg for men, are directly derived from a comprehensive year-long validation study by Whigham et al. [31], whose study demonstrated these average clothing weights to be appropriate for standardizing body weight measurements regardless of outdoor temperature. While these uniform corrections are a pragmatic approach for standardizing measurements, it is acknowledged that individual variations in clothing weight—particularly among older adults who wear diverse garments—may introduce minor measurement error for some participants. These corrections were uniformly applied to all participants to ensure comparability. BMI was calculated as weight (kg) divided by height squared (m^2^). Skin carotenoid score was assessed using the Veggie Meter^®^ (Longevity Link, Salt Lake City, UT, USA). This device uses reflection spectroscopy based on resonance Raman principles to noninvasively quantify dermal carotenoids [32]. Scores are expressed in device-specific units (range, 0–1200), with higher values indicating greater carotenoid accumulation. For each participant, three measurements were obtained from the clean fingertip of the left middle finger (after wiping with a sterile sheet), and the mean value was used in the analysis. The Veggie Meter score has been validated as a reliable biomarker of vegetable and fruit intake [33].

Additionally, dietary intake of food groups and nutrients was assessed using the BDHQ, which has been validated for use in the Japanese population [34,35]. The results of the food group-specific and nutrient intake analyses are presented in the Supplementary Materials. Given the exploratory nature of these analyses and the large number of comparisons, the findings for food group and nutrient intake should be interpreted with caution, acknowledging the increased risk of Type I errors.

In addition to the primary and secondary outcomes, the feasibility and acceptability of the SKN application were evaluated at follow-up using a self-administered questionnaire. The questionnaire included items on the frequency of application use, perceived difficulty and reasons for difficulty, perceived usefulness of individual and team feedback, perceived enjoyment, extent of social interaction through the application, and willingness to continue use.

### 2.6. Statistical Analyses

All analyses followed the intention-to-treat principle and included 29 participants who completed baseline assessment. Between-group differences in outcome measures at 3-month follow-up were assessed using analysis of covariance (ANCOVA), adjusting for baseline values of the respective outcome, age, education status, and antihypertension drug use. A two-sided significance level of 5% was considered statistically significant. All analyses were performed using STATA (version 19.0; StataCorp, College Station, TX, USA).

### 2.7. Use of Generative AI

Generative artificial intelligence (ChatGPT, OpenAI, San Francisco, CA, USA) was used to support language refinement, English–Japanese translation, and improvement of text clarity and structure during manuscript preparation. The tool was not used for the study design, data collection, statistical analysis, or interpretation of the results. All content was written, reviewed, and finalized by the authors.

## 3. Results

### 3.1. Study Participants

Twenty-nine participants were included in the final analysis (11 and 18 from the intervention and control groups, respectively), all of whom completed both baseline and follow-up assessments. The baseline characteristics of the two groups are presented in Table 1.

### 3.2. Primary Outcome

As shown in Table 2, the Japanese Food Guide Spinning Top Score at 3-month follow-up, adjusted for baseline score, age, education status, and antihypertension drug use, was 49.0 (SE = 2.6) and 39.5 (SE = 2.0) in the intervention and control groups, respectively. The adjusted mean difference between groups was +9.5 (95% CI: 2.3 to 16.7), which was statistically significant (*p* = 0.01).

Supplementary Tables S1 and S2 present exploratory findings on food group and nutrient intake. Regarding food group intake (Table S1), the intervention group showed a significantly higher adjusted mean intake of cereals compared to that of the control group (adjusted mean difference = 83.4 g/day, 95% CI: 20.3 to 146.4; *p* = 0.01). Conversely, the intervention group had a significantly lower adjusted mean intake of confectioneries (adjusted mean difference = −29.7 g/day, 95% CI: −51.4 to −7.9; *p* = 0.009). No other food groups showed statistically significant between-group differences.

Regarding nutrient intake (Table S2), several nutrients showed statistically significant between-group differences. The intervention group exhibited significantly higher adjusted mean intakes of potassium (adjusted mean difference = 548 mg/day, 95% CI: 15 to 1076; *p* = 0.04), calcium (adjusted mean difference = 144.8 mg/day, 95% CI: 9.8 to 279.7; *p* = 0.04), Beta-carotene (adjusted mean difference = 2971 μg/day, 95% CI: 1182 to 4760; *p* = 0.002), vitamin K (adjusted mean difference = 146.2 μg/day, 95% CI: 42.0 to 250.4; *p* = 0.008), folate (adjusted mean difference = 79.7 μg/day, 95% CI: 6.2 to 153.2; *p* = 0.04), water-soluble dietary fiber (adjusted mean difference = 1.0 g/day, 95% CI: 0.3 to 1.7; *p* = 0.01), non-soluble dietary fiber (adjusted mean difference = 3.2 g/day, 95% CI: 1.6 to 4.7; *p* < 0.001), and total dietary fiber (adjusted mean difference = 4.2 g/day, 95% CI: 2.0 to 6.5; *p* = 0.001) compared to those of the control group. A trend towards a higher adjusted mean intake of pantothenic acid was also observed in the intervention group (adjusted mean difference = 1.4 mg/day, 95% CI: −0.1 to 2.9; *p* = 0.058). Other nutrients did not show statistically significant between-group differences after ANCOVA adjustment.

### 3.3. Secondary Outcomes

As shown in Table 3, after adjusting for respective baseline values, age, university education, and antihypertension drug use using ANCOVA, no statistically significant between-group differences were observed at 3-month follow-up for any secondary outcomes: body weight (Adjusted Mean Difference = +0.65 kg, 95% CI: −0.06 to 1.37; *p* = 0.07), BMI (Adjusted Mean Difference = +0.26 kg/m^2^, 95% CI: −0.06 to 0.59; *p* = 0.10), skin carotenoid score (Adjusted Mean Difference = +3.58, 95% CI: −29.6 to 36.8; *p* = 0.83), or UCLA loneliness score (Adjusted Mean Difference = −0.18, 95% CI: −1.58 to 1.23; *p* = 0.80).

### 3.4. Feasibility and Acceptability

The feasibility and acceptability of the SKN application were evaluated using a post-intervention questionnaire. Most participants reported using the application once or thrice per week, with only a minority using it daily. Perceived difficulty varied: nearly half of the participants (approximately 45%) felt that the application was relatively difficult to use, whereas a similar proportion found it relatively easy or very easy; those who rated it as difficult were older on average (75.6 years old) than those who rated it as easy (66.0 years old).

Regarding perceived benefits, more than half of the participants considered the activities enjoyable, and >90% found individual feedback useful. Team-level feedback was also perceived as useful by more than half of the participants, although fewer rated it as high as individual feedback. Although only one participant expressed a strong intention to continue using the application, most reported valuing the feedback received.

## 4. Discussion

This study demonstrated that an AI-assisted smartphone application-based intervention significantly improved dietary quality among community-dwelling older adults with frailty. While a non-significant trend towards an increase in body weight was observed (*p* = 0.07), no statistically significant effects were detected for any other secondary outcomes, including BMI, skin carotenoid score, and UCLA loneliness score. Notably, all participants completed the baseline and follow-up assessments, which strengthened the reliability of the findings despite the inherently small sample size and associated limitations in statistical power.

Our primary finding was a significant improvement in overall dietary quality within the intervention group, evidenced by significantly higher Japanese Food Guide Spinning Top Scores at 3-month follow-up compared to those of the control group. Exploratory analyses, interpreted with caution due to the risk of Type I errors, further revealed that this improvement was associated with a higher adjusted mean intake of cereals and a lower adjusted mean intake of confectioneries in the intervention group. This significant improvement in dietary quality can be attributed to the multifaceted nature of the intervention, which aligns with several behavioral science theories. The AI-generated personalized feedback, verified by a dietitian, likely served as a powerful “nudge,” raising participants’ awareness of their dietary choices and facilitating self-regulation, consistent with Nudge Theory [36]. This timely and tailored feedback could have enhanced self-efficacy and autonomous motivation by providing actionable insights and fostering a sense of competence in making healthier choices, as posited by Social Cognitive and Self-Determination Theories. Furthermore, peer interactions through the application, where members viewed, reacted to, and commented on each other’s meal photos, likely promoted observational learning and social support, reinforcing healthy eating behaviors. The dietitian’s role in reviewing AI output and providing group-level advice added a layer of professional validation and guidance to these processes. Adherence to the Japanese Food Guide Spinning Top and consumption of well-balanced meals, including staple, main, and side dishes, are associated with lower odds of frailty [37] and a higher skeletal muscle mass index [38] among Japanese older adults. Overall, our findings strongly emphasize that proactive digital dietary interventions, by leveraging personalized feedback, professional oversight, and peer support, are highly effective for preserving and improving dietary quality in frail older adults, thereby potentially supporting their functional capacity and overall health.

This study examined the feasibility and acceptability of implementing an AI-assisted dietary intervention in older adults with frailty. Overall, the participants evaluated the intervention positively; most found the AI-based feedback useful and the activities enjoyable, although some experienced usability challenges, particularly among older participants. Importantly, all participants completed the 3-month intervention without dropping out, suggesting that the program was feasible and well tolerated, even among individuals with frailty. These findings indicate that although AI-assisted dietary feedback is acceptable and meaningful, digital literacy and user-interface design remain critical considerations for this population. Our results are consistent with those of previous systematic reviews, showing that smartphone applications can improve dietary behaviors in adults [15,39]. Recently, Pala et al. [20] highlighted the growing number of AI-based dietary applications worldwide and the emerging validation efforts. Nevertheless, few studies have targeted older adults [23], and evidence among those with frailty remains extremely limited. In this context, our study provides preliminary evidence that an AI-assisted dietary application can be implemented among community-dwelling older adults with frailty with full participation maintained throughout the intervention period. Although usability challenges were evident, the generally positive responses suggest that with further refinement and user-centered adaptation, such tools can become practical components of nutritional care for this vulnerable population.

This study has several limitations. First, the sample size was small, which limited the statistical power and the generalizability of our findings and restricted our ability to detect more subtle but clinically meaningful effects. Future research with larger, adequately powered samples—ideally determined through a priori power calculations—is warranted to confirm and extend these preliminary observations. Second, this study used a quasi-experimental design without random assignment, which limits the generalizability and scalability of the findings and increases the risk of selection bias due to pre-existing group differences, partially attributable to the non-randomized assignment criteria where staff assigned participants with higher smartphone skills to the intervention group and those unable to attend baseline assessments for the intervention group were assigned to the control group. These factors likely contributed to the observed baseline imbalances. To mitigate these limitations, we used ANCOVA for statistically adjusting for important baseline characteristics, including age, education status, and antihypertension drug use. However, even with these adjustments, the inherent limitations of a quasi-experimental design mean that direct causal inferences should be made with caution. Additionally, participants were limited to current smartphone users who were relatively independent community-dwelling older adults. This inherent selection excludes the frailest elderly, who are often disproportionately affected by the digital divide and may lack access to or proficiency with smartphones. Therefore, the applicability of these results to more vulnerable or digitally unaccustomed older populations may be limited. Third, frailty status was assessed using the KCL, a self-administered instrument that may be subject to self-report bias and potential misclassification. A fourth limitation relates to the assessment of body weight. Although a pragmatic uniform subtraction of 0.8 kg was applied for women to account for clothing weight, this approach inherently introduces potential measurement error. Clothing weight can vary considerably among individuals, particularly in older adults who may choose diverse types of garments based on preference, comfort, or health needs. As a result, this uniform adjustment may not reflect true body weight for all participants and could have led to slight inaccuracies in weight-related measurements and analyses. Future studies may consider more individualized methods for accounting for clothing weight, such as weighing garments separately or using standardized garment weight tables. Fifth, there was a potential risk of contamination between the intervention and control groups. Because participants lived in the same municipality and may have participated in community activities or informal group chats, “spillover effects” could have occurred, whereby information or influence from the intervention group reached the control group. The study did not include a monitoring system for cross-group communication, and thus this form of contamination cannot be ruled out. Such unintended information exchange may have attenuated the observed differences between groups. Sixth, we did not quantitatively assess the intensity of application engagement, such as frequency of use or responsiveness to feedback, which limits our ability to evaluate dose–response relationships. Future studies should incorporate objective usage metrics to better characterize engagement patterns. Finally, the follow-up period was relatively short (3 months), limiting our ability to assess the long-term sustainability of the observed effects. Despite these limitations, this study has several strengths. All participants completed both baseline and follow-up assessments, demonstrating the feasibility of maintaining engagement with a digital dietary intervention even among older adults with frailty. Moreover, the AI-based image-recognition algorithm embedded in the application was validated in advance against trained dietitians and showed acceptable accuracy before implementation, ensuring feedback reliability.

## 5. Conclusions

In this quasi-experimental study, we evaluated an AI-assisted smartphone application-based intervention designed to improve dietary quality among community-dwelling older adults identified as frail. Our findings indicate that the intervention significantly improved overall dietary quality over a three-month period, suggesting the feasibility and preliminary efficacy of a technology-enabled approach to support healthier eating habits in this vulnerable population. While the primary outcome demonstrated significant improvement, no statistically significant effects were observed for most secondary outcomes, including BMI, skin carotenoid scores, and UCLA loneliness scores. Although participant engagement and high completion rates underscore the practical feasibility of the intervention, these results must be interpreted with caution.

The relatively small sample size, the quasi-experimental design lacking random assignment, and the short follow-up period limit the ability to draw definitive causal inferences or to generalize the findings to broader older adult populations—particularly those without access to smartphones or with limited digital literacy. In conclusion, this study provides initial evidence that an AI-assisted smartphone application may serve as a promising tool for enhancing dietary quality among frail, community-dwelling older adults. Future research employing larger, randomized controlled trials with longer follow-up periods is needed to confirm these preliminary findings, evaluate long-term sustainability, and assess the broader impact on health outcomes across diverse older adult groups.

## Figures and Tables

**Table 1:** Baseline Characteristics of the Study Participants.

	Intervention (*n* = 11)		Control (*n* = 18)	
Age, years (mean, SD)	74.6	(5.5)	79.2	(6.5)
BMI, kg/m^2^ (mean, SD)	21.9	(2.9)	21.9	(1.6)
Skin carotenoid score (mean, SD)	387.6	(109.4)	414	(127.5)
Women (*n*, %)	11	(100.0)	17	(94.4)
Live together (*n*, %)	8	(72.7)	13	(72.2)
Employed (*n*, %)	2	(18.2)	5	(27.8)
Highest educational attainment (*n*, %)				
Junior high school	0	(0.0)	2	(11.1)
High school	2	(18.2)	8	(44.4)
College	4	(36.4)	7	(38.9)
University	5	(45.5)	1	(5.6)
Current living situation (*n*, %)				
Difficult	0	(0.0)	0	(0.0)
Average	9	(81.8)	13	(72.2)
Comfortable	2	(18.2)	5	(27.8)
Alcohol drinking frequency (*n*, %)				
Rarely	7	(63.6)	15	(83.3)
1–4 days/week	1	(9.1)	0	(0.0)
Almost every day	3	(27.3)	3	(16.7)
Smoking status (*n*, %)				
Nonsmoker	7	(63.6)	16	(88.9)
Past smoker	4	(36.4)	1	(5.6)
Current smoker	0	(0.0)	1	(5.6)
Self-rated health (*n*, %)				
Poor	0	(0.0)	2	(11.1)
Fair	2	(18.2)	4	(22.2)
Good	9	(81.8)	12	(66.7)
Antihypertension drug use (*n*, %)	1	(9.1)	9	(50.0)
Antidiabetes drug use (*n*, %)	0	(0.0)	0	(0.0)
Antihyperlipidemia drug use (*n*, %)	5	(45.5)	7	(38.9)
History of stroke (*n*, %)	0	(0.0)	0	(0.0)
History of heart disease (*n*, %)	1	(9.1)	1	(5.6)
History of kidney disease (*n*, %)	0	(0.0)	1	(5.6)

BMI, body mass index; SD, standard deviation.

**Table 2:** Japanese Food Guide Spinning Top Scores at 3-Month Follow-up (Adjusted by ANCOVA).

	Baseline Mean (SD)		Adjusted Mean (SE) at 3-Month Follow-Up ^1^		Between-Group Difference (95% CI) ^2^		*p* Value ^2^
Intervention	43.7	(9.8)	49.0	(2.6)	9.5	(2.3, 16.7)	0.01
Control	45.6	(6.8)	39.5	(2.0)			

^1^ Adjusted for baseline Japanese Food Guide Spinning Top Score, age, education status, and antihypertension drug use. ^2^ *p*-value for between-group difference from ANCOVA. ANCOVA, analysis of covariance; SE, standard error; CI, confidence interval.

**Table 3:** Secondary Outcomes at 3-Month Follow-up (Adjusted by ANCOVA).

	Baseline Mean (SD)		Adjusted Mean (SE) at 3-Month Follow-Up ^1^		Between-Group Difference (95% CI) ^2^		*p* Value ^2^
**Weight**							
Intervention	50.0	(6.2)	50.1	(0.3)	0.65	(−0.06, 1.37)	0.07
Control	51.3	(6.6)	49.4	(0.2)			
**BMI**							
Intervention	21.9	(2.9)	21.8	(0.1)	0.26	(−0.06, 0.59)	0.10
Control	21.9	(1.6)	21.5	(0.1)			
**Skin carotenoid score**							
Intervention	387.6	(109.4)	425.3	(12.0)	3.58	(−29.6, 36.8)	0.83
Control	414.0	(127.5)	421.7	(9.1)			
**UCLA loneliness score**							
Intervention	6.91	(1.14)	5.61	(0.49)	−0.18	(−1.58, 1.23)	0.80
Control	5.33	(1.53)	5.79	(0.36)			

^1^ Adjusted for respective baseline outcome score, age, education status, and antihypertension drug use. ^2^ *p*-value for between-group difference from ANCOVA.

## Data Availability

The data supporting the findings of this study are not publicly available owing to ethical and privacy restrictions. Individual-level data contained information that could compromise the privacy of the research participants. Therefore, data sharing was not included in the informed consent form, which was approved by the institutional ethics committee. Aggregated or anonymized data may be made available by the corresponding author upon reasonable request and with the approval of the ethics committee.

## Supplementary Materials

The following supporting information can be downloaded from https://www.mdpi.com/article/10.3390/geriatrics10060160/s1, Table S1: Changes in food group intake from baseline to 3-month follow-up; Table S2: Changes in nutrient intake from baseline to 3-month follow-up.

## Abbreviations

The following abbreviations are used in this manuscript:

BMI	Body mass index
ICT	Information and communication technology
AI	Artificial intelligence
KCL	Kihon Check List
SKN	Shokuji Kōkan Nikki
