# Supplementary material for: An Artificial Intelligence-Assisted Smartphone Application for Improving Dietary Quality Among Frail Older Adults: A Quasi-Experimental Study
Source: Geriatrics (Basel). 2025 Dec 4;10(6):160. doi: 10.3390/geriatrics10060160 (PMC12732455; doi:10.3390/geriatrics10060160)
Supplement: Supplementary file 1 [file geriatrics-10-00160-s001.zip › geriatrics-3980287-supplementary.pdf]

Table S1 Food group intake at 3-month follow-up (Adjusted by ANCOVA)

	Intervention group				Control group						
	Baseline Mean (SD)		Adjusted Mean (SE) at 3-month Follow-up[1]		Baseline Mean (SD)		Adjusted Mean (SE) at 3-month Follow-up[1]		Between-Group Difference (95% CI)[2]		P value[2]
Intake (g/day)											
Cereals	334.8	(165.2)	346.9	(23.4)	296.6	(113.6)	263.4	(17.9)	83.4	(20.3, 146.4)	0.01
Potatoes	60.0	(45.4)	75.5	(13.1)	69.1	(65.8)	64.9	(10.0)	10.6	(-24.5, 45.7)	0.54
Sugars and sweeteners	3.7	(2.1)	6.5	(0.9)	9.5	(6.2)	6.0	(0.6)	0.5	(-1.9, 2.9)	0.67
Pulses	82.4	(40.8)	98.3	(10.3)	91.0	(77.0)	77.4	(7.9)	21.0	(-6.6, 48.6)	0.13
Green and yellow vegetables	128.1	(72.5)	196.7	(18.7)	174.7	(79.8)	165.5	(14.3)	31.2	(-19.4, 81.8)	0.22
Other vegetables	179.4	(94.3)	238.5	(18.2)	247.2	(133.8)	199.6	(13.9)	39.0	(-10.4, 88.4)	0.12
Fruits	164.5	(98.0)	221.4	(35.3)	267.5	(161.8)	229.1	(26.9)	-7.7	(-103.6, 88.2)	0.87
Fish and shellfish	87.7	(57.1)	113.9	(18.6)	161.0	(186.2)	132.8	(14.3)	-18.9	(-69.0, 31.1)	0.44
Meat	83.6	(40.8)	113.1	(15.3)	139.5	(108.0)	92.0	(11.7)	21.1	(-20.2, 62.4)	0.30
Eggs	43.3	(18.2)	58.0	(5.5)	57.6	(28.2)	44.9	(4.2)	13.2	(-1.6, 28.0)	0.08
Milk and dairy products	154.1	(106.2)	196.0	(27.0)	204.2	(109.2)	153.5	(20.7)	42.5	(-30.3, 115.3)	0.24
Oils	12.2	(4.6)	11.8	(1.8)	11.9	(5.5)	12.3	(1.4)	0.4	(-5.4, 4.6)	0.87
Confectioneries	83.9	(71.8)	63.8	(8.1)	81.5	(51.8)	93.4	(6.2)	-29.7	(-51.4, -7.9)	0.009
Beverages	563.6	(308.1)	686.9	(78.6)	669.1	(326.4)	654.4	(60.4)	32.5	(-178.7, 243.7)	0.75
Seasonings and spices	243.8	(189.7)	253.9	(27.8)	203.1	(106.5)	187.1	(21.3)	77.8	(-8.1, 141.6)	0.08

[1]Adjusted for respective baseline outcome score, age, education status, and antihypertension drug use.

[2]P-value for between-group difference from ANCOVA.

Table S2 Nutrients intake at 3-month follow-up (Adjusted by ANCOVA)

	Intervention group				Control group				Between-Group Difference (95% CI)[2]		P value[2]
	Baseline Mean (SD)		Adjusted Mean (SE) at 3-month Follow-up[1]		Baseline Mean (SD)		Adjusted Mean (SE) at 3-month Follow-up[1]				
Energy (kcal/day)	1943	(638)	2185	(100)	2253	(871)	2016	(76)	169	(-98, 437)	0.21
Protein (g/day)	79.7	(30.3)	99.3	(6.6)	112	(72.1)	90.1	(5.1)	9.2	(-8.7, 27.1)	0.30
Animal protein (g/day)	46.4	(17.5)	61.6	(5.7)	76.4	(64.8)	57.1	(4.4)	4.4	(-11.0, 19.8)	0.56
Vegetable protein (g/day)	33.3	(13.7)	37.3	(1.9)	35.6	(12.8)	33.2	(1.5)	4.2	(-1.1, 9.4)	0.11
Fat (g/day)	62.5	(23.5)	72.3	(5.5)	79.5	(41.1)	68.6	(4.2)	3.7	(-11.2, 18.6)	0.61
Animal fat (g/day)	27.4	(10.3)	34.7	(3.1)	42	(31.5)	32.2	(2.3)	2.4	(-5.8, 10.7)	0.55
Vegetable fat (g/day)	35.1	(15.5)	36.9	(2.8)	37.5	(13.6)	36.8	(2.1)	0.1	(-7.4, 7.6)	0.97
Carbohydrate (g/day)	242.7	(96.7)	261.1	(13)	260.1	(78.4)	242.1	(10)	19	(-15.9, 53.9)	0.27
Sodium (mg/day)	4405	(1732)	5206	(328)	5714	(2560)	4803	(251)	403	(-481, 1287)	0.36
Potassium (mg/day)	3071	(1277)	4000	(196)	4079	(1987)	3454	(150)	548	(15, 1076)	0.04
Calcium (mg/day)	670.1	(346.3)	870.1	(50)	889.1	(522.1)	725.4	(38.4)	144.8	(9.8, 279.7)	0.04
Magnesium (mg/day)	301.8	(118.1)	371.2	(19.3)	378.5	(196)	324.9	(14.8)	46.3	(-5.6, 98.2)	0.08
Phosphorus (mg/day)	1234	(482)	1525	(97)	1687	(1058)	1360	(75)	165	(-97, 427)	0.21
Iron (mg/day)	9.4	(3.9)	12	(0.6)	12.4	(6.3)	10.7	(0.5)	1.2	(-0.4, 3.0)	0.16
Zinc (mg/day)	9.2	(3.1)	10.9	(0,6)	11.9	(6.3)	9.5	(0.5)	1.4	(-0.3, 3.2)	0.10
Copper (mg/day)	1.3	(0.5)	1.5	(0.1)	1.6	(0.7)	1.4	(0.1)	0.2	(-0.1, 0.4)	0.16
Manganese (mg/day)	3.0	(1.3)	3.6	(0.3)	3.6	(1.5)	3.4	(0.2)	0.2	(-0.5, 0.9)	0.51
Retinol (μg/day)	502.4	(305.4)	484	(105.5)	1048.5	(1584)	594.9	(81.1)	-110.9	(-394.7, 172.9)	0.43
Beta-carotene (μg/day)	4457	(2811)	8050	(659)	6142	(3001)	5079	(503)	2971	(1182, 4760)	0.002
Retinol equivalents (μg/day)	878	(438)	1114	(143)	1566	(1647)	1051	(110)	62	(-324, 449)	0.74

Vitamin D (µg/day)	17.6	(10.8)	22.7	(3.4)	28.5	(33.9)	22.8	(2.6)	-0.2	(-9.2, 8.9)	0.97
Alpha-tocopherol (mg/day)	8.8	(4.0)	11.3	(0.9)	12	(6.5)	10.5	(0.7)	0.9	(-1.6, 3.4)	0.27
Vitamin K (µg/day)	433.8	(212.3)	604.8	(38.7)	512.4	(268.9)	458.6	(29.7)	146.2	(42.0, 250.4)	0.008
Vitamin B1 (mg/day)	0.9	(0.4)	1.1	(0.1)	1.3	(0.6)	1	(0.1)	0.1	(-0.04, 0.3)	0.13
Vitamin B2 (mg/day)	1.5	(0.6)	1.9	(0.1)	2.2	(1.1)	1.7	(0.1)	0.2	(-0.1, 0.5)	0.15
Niacin (mg/day)	19.2	(6.8)	24.8	(2.1)	28.4	(20.7)	23.2	(1.6)	1.6	(-4.0, 7.2)	0.56
Vitamin B6 (mg/day)	1.5	(0.6)	1.9	(0.1)	2.1	(1.3)	1.7	(0.1)	0.2	(-0.1, 0.5)	0.18
Vitamin B12 (µg/day)	10.3	(6.1)	13.2	(1.9)	18.4	(18.8)	15.4	(1.5)	-2.2	(-7.4, 3.0)	0.39
Folate (µg/day)	399.7	(195.4)	546.2	(27.1)	560.6	(272.9)	466.4	(20.8)	79.7	(6.2, 153.2)	0.04
Pantothenic acid (mg/day)	7.7	(2.6)	9.7	(0.5)	10.5	(5.3)	8.3	(0.4)	1.4	(-0.1, 2.9)	0.058
Vitamin C (mg/day)	141.5	(80.8)	202.3	(15.9)	215.2	(92.6)	178.5	(12.1)	23.8	(-19.6, 67.1)	0.27
Saturated fatty acids (g/day)	16.2	(6.3)	18.8	(1.4)	21.4	(11)	17.7	(1)	1	(-2.6, 4.7)	0.57
Monounsaturated fatty acids (g/day)	22.5	(8.5)	25.7	(2.1)	28.4	(13.9)	24.5	(1.6)	1.2	(-4.5, 6.9)	0.67
Polyunsaturated fatty acids (g/day)	15.3	(5.7)	17.7	(1.6)	18.5	(10.4)	16.8	(1.2)	0.9	(-3.3, 5.2)	0.66
Cholesterol (mg/day)	465.8	(163.3)	582.4	(49.7)	673.8	(395.5)	521.2	(38)	61.2	(-73.0, 195.4)	0.36
Water-soluble dietary fiber (g/day)	4.1	(1.8)	5.1	(0.3)	4.8	(2)	4.1	(0.2)	1	(0.3, 1.7)	0.01
Non-soluble dietary fiber (g/day)	10.6	(4.6)	14	(0.6)	12.8	(5.2)	10.9	(0.4)	3.2	(1.6, 4.7)	<0.001
Total dietary fiber (g/day)	15.2	(6.4)	19.8	(0.8)	18	(7.3)	15.6	(0.6)	4.2	(2.0, 6.5)	0.001
Salt (g/day)	11.1	(4.4)	13.2	(0.8)	14.5	(6.5)	12.1	(0.6)	1	(-1.2, 3.2)	0.36

[1]Adjusted for respective baseline outcome score, age, education status, and antihypertension drug use.

[2]P-value for between-group difference from ANCOVA.
